# Characterization and Quantification of Depletion and Accumulation Layers in Solid‐State Li^+^‐Conducting Electrolytes Using In Situ Spectroscopic Ellipsometry

**DOI:** 10.1002/adma.202100585

**Published:** 2021-05-06

**Authors:** Leon Katzenmeier, Leif Carstensen, Simon J. Schaper, Peter Müller‐Buschbaum, Aliaksandr S. Bandarenka

**Affiliations:** ^1^ Physics of Energy Conversion and Storage Department of Physics Technische Universität München James‐Franck‐Str. 1 Garching 85748 Germany; ^2^ Bayerisches Zentrum für Angewandte Energieforschung Magdalene‐Schoch‐Str. 3 Würzburg 97074 Germany; ^3^ Lehrstuhl für Funktionelle Materielien Physik‐Department Technische Universität München James‐Franck‐Str. 1 Garching 85748 Germany; ^4^ Heinz Maier‐Leibnitz Zentrum (MLZ) Technische Universität München Lichtenbergstr. 1 Garching 85748 Germany; ^5^ e‐conversion Excellence Cluster Lichtenbergstr. 4 Garching 85748 Germany

**Keywords:** accumulation layers, solid Li‐ion conductors, solid‐state electrolytes, space charge layer, spectroscopic ellipsometry

## Abstract

The future of mobility depends on the development of next‐generation battery technologies, such as all‐solid‐state batteries. As the ionic conductivity of solid Li^+^‐conductors can, in some cases, approach that of liquid electrolytes, a significant remaining barrier faced by solid‐state electrolytes (SSEs) is the interface formed at the anode and cathode materials, with chemical instability and physical resistances arising. The physical properties of space charge layers (SCLs), a widely discussed phenomenon in SSEs, are still unclear. In this work, spectroscopic ellipsometry is used to characterize the accumulation and depletion layers. An optical model is developed to quantify their thicknesses and corresponding concentration changes. It is shown that the Li^+^‐depleted layer (≈190 nm at 1 V) is thinner than the accumulation layer (≈320 nm at 1 V) in a glassy lithium‐ion‐conducting glass ceramic electrolyte (a trademark of Ohara Corporation). The in situ approach combining electrochemistry and optics resolves the ambiguities around SCL formation. It opens up a wide field of optical measurements on SSEs, allowing various experimental studies in the future.

## Graphical abstract

Solid‐state electrolytes are one of the few battery technologies for next‐generation batteries. However, the high interface resistance in contact with electrode materials is still largely unexplained. Spectroscopic ellipsometry combined with in situ electrochemistry reveals highly asymmetric space charge layer occurrence. It opens a new study field to investigate and quantify the charge layer formation in solid‐state electrolytes by optical means.

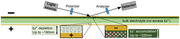

## 1 Introduction

The ever increasing demand for energy storage has led to the lithium‐ion battery (LiB) invention with its first commercialization in 1991.^[^ ^1^ ^]^ After 30 years, this battery technology still dominates the market today with its ubiquitous use in mobile phones and battery electric vehicles.^[^ ^2^ ^]^ However, the holy grail of anode materials, metallic Lithium, is thought of as being inapplicable to conventional LiBs as the dendrite formation cannot be mitigated so far in a system using liquid electrolytes.^[^ ^3^ ^]^ All‐solid‐state batteries could be a possible solution,^[^ ^4^ ^]^ in which a solid‐state electrolyte (SSE) replaces the liquid electrolyte and the separator of a conventional battery, posing an impenetrable barrier to lithium dendrites.^[^ ^5^ ^]^ The resulting thin‐film battery should then have a much higher gravimetric and volumetric energy density, provided that the SSE is sufficiently thin.^[^ ^6^ ^]^ However, a stack of solid materials introduces a set of different challenges, mainly at the interfaces. The issues of mechanical instability^[^ ^7^ ^]^ and interphase formation^[^ ^8^ ^]^ leading to a high interface resistance need to be addressed.^[^ ^9^ ^]^ One can mitigate these problems by using an additional layer^[^ ^10^ ^]^ or controlled formation of Li‐ion conducting passivation layers.^[^ ^11^ ^]^ Mitigating these problems leaves the high interface resistance's physical origin unexplained. Space charge layer (SCL) formation in SSEs with a single mobile charge species has been suggested as early as 1981.^[^ ^12^ ^]^ Similar to the double layer formation in liquids, the SCL forms at any interface between two materials with different (electro‐)chemical potentials. The prime example is the solid‐solid interface between electrode and electrolyte.

In liquid electrolytes, the interface of two materials with different ionic and electronic conductivities is well understood.^[^ ^13^ ^]^ The theory of the so‐called electrochemical double layers has been under development for more than 100 years with the description of a very compact (sub‐nm) Helmholtz layer and a diffuse layer (up to 50 nm) reaching further into the electrolyte but with a lower ion concentration change.^[^ ^14^ ^]^

The same layer structure was found when investigating the electrochemical nature of these SCLs in blocking conditions, with no interfacial Li^+^ transfer, as elucidated in previous work.^[^ ^15^ ^]^ Upon applying a bias potential to the SSE under blocking conditions, the bulk of the electrolyte will be shielded by two oppositely charged layers formed at the interfaces between electrodes and electrolyte. Adjacent to the negatively biased electrode, the only mobile species (Li^+^) will accumulate on vacant lattice sites to form an accumulation layer. In the following, we call this a “positive SCL” (p‐SCL). Hence, the vacancies’ density creates a boundary condition to the maximum concentration of Li^+^ in such a layer. As global charge neutrality must hold for the electrolyte in blocking conditions, the p‐SCL must have a counterpart, that is, a Li^+^‐depletion layer. This “negative SCL” (n‐SCL) will form at the SSE's positively biased side. Here, the concentration of mobile Li^+^ forms the boundary condition, as this is the maximum depletion achievable. It is essential to notice that the concentration of free vacancies and mobile Li^+^ does not have to be equal, leading to a possible asymmetry of both SCL thickness and concentration. However, the total charge depleted and accumulated has to match if the electrolyte is under blocking conditions.

The advancement of SSE's led researchers working on battery technology to investigate this issue experimentally and theoretically. However, the conclusions are contradictory and range from: i) layers of a few hundred nanometers thickness and significant impact^[^ ^16^, ^17^ ^]^ to ii) negligible impact and only a single nanometer thickness.^[^ ^18^, ^19^ ^]^

Within the class of solid electrolytes, oxides excel at atmospheric stability and processability, making them an attractive model material system.^[^ ^20^ ^]^ A commonly used glass‐ceramic of this type is the lithium‐ion‐conducting glass ceramic (LICGC), a trademark of Ohara Corporation, a polycrystalline material with main crystalline compounds of Li_2_O–Al_2_O_3_–SiO_2_–P_2_O_5_–TiO_2_–GeO_2,_ which is the material studied in this work. Although it is such a well‐studied material, the exact material parameters, for example, the cation and vacancy densities, still remain unknown. Knowledge about these parameters would greatly benefit theoretical work and could be used to validate experimental findings mathematically.

**Figure 1:**
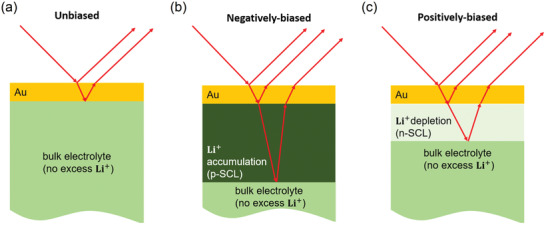
Schematic representation of the sample set‐up consisting of the solid‐state electrolyte (green) and the gold electrode (yellow). The red arrows schematically mark the light path during the ellipsometer measurement: a) layer model without bias potential, b) with an additional Li^+^‐accumulation layer (dark green), and c) with an Li^+^‐depletion layer (light green).

In this work, we show that an optical method, spectroscopic ellipsometry, combined with a semi‐empirical model for the optical properties of the SCL can be used to elucidate the formation of asymmetric SCLs. It is important to note, that the experimental resolution (nm) does not allow to account for the compact double layer (sub‐nanometer). **Figure** **1** shows the layer structure of the charge accumulation and depletion depending on the bias for only the top electrode for three cases. Figure 1a) corresponds to no applied potential, Figure 1b) corresponds to the negative applied potential, and Figure 1c) corresponds to the positive applied potential.

The in situ approach allows comparison of these findings to previously published work.^[^ ^15^ ^]^ Furthermore, the exact concentration changes in and the thickness of these layers are determined with fits using an effective medium approximation (EMA). Similar to the eye visible, optical changes of Li‐intercalation into a graphite anode,^[^ ^21^ ^]^ we assumed that the presence and absence of Lithium do change the optical properties in a way detectable by the ellipsometer. Significant changes of the refractive index and extinction coefficient in the visible range upon Li^+^ de‐/intercalation were quantified for lithium manganese oxide.^[^ ^22^ ^]^

## 2 Results and Discussion

**Figure 2:**
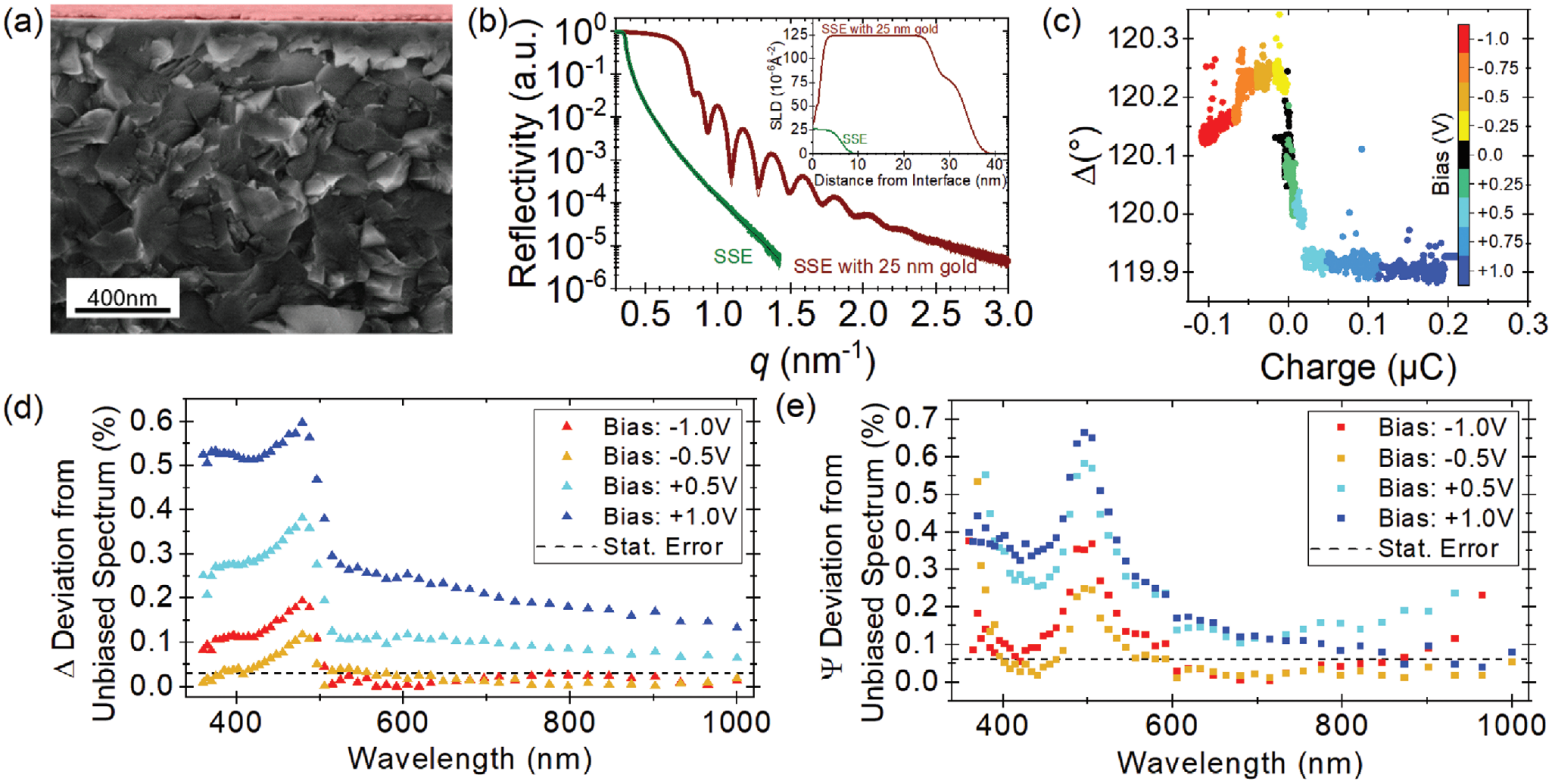
a) Scanning electron microscopy image of the electrolyte in a cross‐sectional view with the gold electrode marked in red. b) XRR and SLD (embedded diagram) of the SSE with (red) and without gold electrode (green). c) The dependency of the ellipsometric angle Δ on the electric charge at different bias potentials. d,e) Changes to the unbiased spectrum in Δ (d) and Ψ (e) as a function of the wavelength at different bias potentials. The average statistical errors are given as a dashed black line in both plots to show the sensitivity of the ellipsometer.

The SSE's polycrystalline nature can be observed in the scanning electron microscopy image (**Figure** **2**a), with grain sizes of a few hundred nanometers. The interface toward the blocking electrode (marked red in Figure 2a) is planar and smooth, indicating a surface roughness below ten nanometers. Knowledge of the physical layer structure is key to building an optical model, and surface roughness is generally considered an obstacle for building quantitative ellipsometry fits.^[^ ^23^ ^]^ Therefore, the thickness of the gold electrode and the interface roughness are investigated using X‐ray reflectivity (XRR) measurements (Figure 2b). As can be observed in the scattering length distribution (SLD) (inset in Figure 2b) resulting from the analysis of the XRR data, the gold surface and the gold/electrolyte interface show a non‐negligible roughness and mixing. The bare electrolyte (SLD_SSE_ = (25.773 ± 0.001) × 10^–6^ Å^–2^) is fitted with the Motofit^[^ ^24^ ^]^ plugin for Igor, while a surface layer (SLD_SSE surface_ = (25.217 ± 0.006) × 10^–6^ Å^–2^) with a thickness of (5.1 ± 0.1) nm and roughness of (1.5 ± 0.1) nm is present. Using a three‐layer model on top of the bulk SSE (SSE/Au‐interface, Au, Au/air‐surface) for the XRR of the Au/SSE sample, the SSE/Au‐interface is determined to be less than 2 nm thick, which is in good agreement with the roughness of the SSE surface layer. The best fit for the SSE/Au‐interface layer is achieved with a thickness of (0.5 ± 0.8) nm and a roughness of (0.9 ± 0.2) nm. The thickness of the Au layer (SLD_Au_ = 124.0 × 10^–6^ Å^–2^) is fit to (24.9 ± 0.2) nm with a roughness of (1.3 ± 0.1) nm, and the Au/air surface layer (SLD_Au/air_ = (81 ± 5) × 10^–6^ Å^–2^) has a thickness of (7.4 ± 0.5) nm with a roughness of (2.2 ± 0.2) nm. The surface layer found on the bare SSE cannot be resolved with evaporated Au on top due to the high contrast of Au compared to very small SLD difference between the surface layer and bulk SSE. At the SSE–(interlayer)–lithium electrode interfaces, alloy formation of Li–Ge, Li–In, Li–Al, Li–Pt, and Li–Au is reported in the literature,^[^ ^25^ ^]^ but the structure of the SSE affects the type of species forming at the interface.^[^ ^26^ ^]^ Without a lithium electrode present in our sample, it is unlikely that an alloy is forming at the SSE–Au interface.

As SSE optics are not frequently investigated, and optical properties are largely unknown, UV–vis transmission measurements are performed on both pure SSE and Au/SSE samples (Figure S1, Supporting Information). The transmission spectrum of the Au/SSE/Au sample later used for in situ experiments, shows ≈3.8% transmission for wavelengths above 500 nm. While no transmission spectra for the Au/SSE system are known in the literature, thermally evaporated Au thin films have shown similar transmission values in the investigated range.^[^ ^27^ ^]^ The penetration of light through the Au layer into the SSE is a prerequisite for any ellipsometric investigation of the SCL underneath the Au electrode. In a spectroscopic ellipsometry measurement of the bare SSE at an angle of incidence (AOI) of 50° shown in Figure S2, Supporting Information, the ellipsometric angles Ψ and Δ follow a well‐defined trend. A Cauchy model is used to fit the dispersion relation (parameters in Figure S2b, Supporting Information) and forms the baseline for the SSE's optical description. When an Au layer is added to the SSE, the model includes the bare SSE, the Au, and an additional mixing layer accounting for the interface roughness. The spectroscopic measurement of the Au/SSE is shown in Figure S2c, Supporting Information. This completes the description of the sample in electrochemical equilibrium.

With these prerequisites in mind, we link electrochemistry and ellipsometry in situ. To show that changes in the electrochemical condition of the sample can be correlated with a change of optical properties, the ellipsometric angles Ψ and Δ are recorded while applying a staircase potential bias between −1 and 1 V. The in situ approach allows for simultaneous measurement of the current flow, which is integrated over time to give the amount of charge. Figure 2c shows the correlation of Δ and charge, which indicates that the formation of an additional layer at the Au/SSE interface can be observed with monochromatic ellipsometry (λ  = 658 nm, AOI 65°). While the monochromatic in situ observation of the ellipsometric angles shows that the impact of the electrochemistry can be measured using ellipsometry, spectroscopic measurements are required to fit a quantitative model to the data.

The elemental distribution for all constituents is determined with energy‐dispersive X‐ray (EDX) measurements and independent of the bias potentials (see Figure S3, Supporting Information). This rules out that any other stoichiometric change at the Au/SSE interface causes the probed changes in optical parameters.

The changes in spectroscopic ellipsometry angles under the application of a bias potential with respect to the 0 V baseline are shown in Figure 2d,e. The model to fit the spectroscopic ellipsometry data (see Figure S4, Supporting Information) consists of dispersion relations and a thickness for each layer, as sketched in Figure 1. The model Au‐Gold_Q‐Sense‐Quarz to describe the Au layer is part of the EP4Model software, which only leaves the thickness as a fitting parameter. The model for the bare SSE is the Cauchy model as described in Figure S2, Supporting Information, which is a semi‐empirical description. The SCL is modeled by an EMA, a common tool in optics. In an EMA, the dispersion relation of a host material, here the SSE described by the Cauchy model, is mixed with that of a guest material (here: Li^+^). The Bruggemann model for EMAs is based on ellipsoidal inclusions of a phase with different optical properties.^[^ ^28^ ^]^ The SCL is described by the mixing coefficient *c*_SCL_ (fraction of Li^+^ in SSE) and the layer thickness *d*_SCL_, which are two degrees of freedom of the final layer model fitted to the ellipsometric spectra obtained at different bias potentials. As the bulk already contains a certain amount of Li^+^, negative concentration changes are allowed to account for the depletion of Li^+^. Therefore, the changes in Li^+^ concentration are measured as a deviation from the bulk concentration in vol%. The change of the Li^+^ concentration directly impacts the optical properties of the mixed layer, as shown in Figure S5, Supporting Information.

It is important to notice that the bulk electrolyte is mathematically represented as a substrate within the model, which leads to an infinite layer thickness. Practically, this means that only the interface closer to the incident light beam is present in the model, an assumption based on the ellipsometer's focus point. Thus, for negative bias potentials, the p‐SCL is observed, whereas, for positive bias potentials, the n‐SCL is observed, as shown in Figure 1.

While polarization of the electrolyte can cause a change in optical properties,^[^ ^29^ ^]^ this effect can be ruled out based on two arguments: i) a polarization should cause a symmetric change in isotropic materials and ii) the potential drop in case of SCLs only happens in the vicinity of the electrodes and therefore the bulk stays unpolarized.

**Figure 3:**
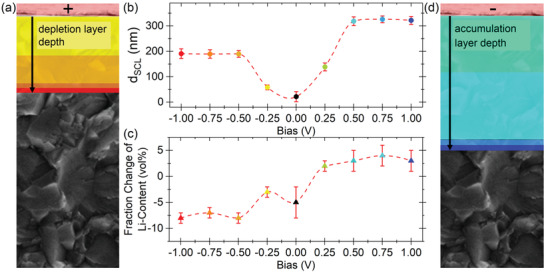
a,d) Color‐coded representation of the SCL growth of the n‐SCL (a) and the p‐SCL (d) at different bias potentials. b) Layer thickness and c) change in Li^+^ concentration compared to the bulk concentration as a function of the bias potential. The red lines in (b) and (c) are auxiliary lines for better visual guidance.

The results of the model fit for the SCL to the spectroscopic ellipsometry data under different bias conditions are shown in **Figure** **3**. The small parameter errors <10% relative to absolute change >100% (*d*_SCL_, *c*_SCL_) and the RMSE <11.5% (see Table S1, Supporting Information) prove the mathematical validity of this approach and the model. As no literature precedent exists to our knowledge, future work can replace the semi‐empirical description with a physically motivated model.

Noticeably, the changes in Li^+^ concentration *c*_SCL_ and layer thickness *d*_SCL_ show a highly asymmetric behavior. The n‐SCL shows a more significant change in concentration, together with a thinner thickness than the p‐SCL, which matches the expectation of an asymmetric formation of SCLs in SSE. To further validate the model, it is also used to fit the 0 V ellipsometric spectrum yielding a vanishing layer thickness and a concentration with a large error.

As expected, the concentrations reach an upper and lower limit for the corresponding polarizations, which is in good agreement with theoretical considerations of boundary conditions of the SSE's crystal lattice. The maximum concentration change is (−8 ± 1) vol% and (+4 ± 2) vol% for the n‐SCL and the p‐SCL, respectively.

For higher polarizations, above an absolute value of 0.5 V, the SCLs grow into the electrolyte to hold the additional charges. The extension of the n‐SCL perpendicular to the surface reaches a maximum of (190 ± 19) nm at −1 V. The much less concentrated p‐SCL is growing to a maximum thickness of (321 ± 15) nm at 1 V.

Comparability of the different measurements is ensured by the fact that the illuminated spot is the same throughout all spectra. Hence, we are not looking at other areas of the material but only at its electrochemical state in situ. The calculated n‐SCL thickness is in good agreement with the order of magnitude found using impedance spectroscopy in earlier work.^[^ ^15^ ^]^

The absolute number of charges *n*_Li,excess_ can be calculated using Equation (1) to be of the order of 1 µmol Li^+^ (see Table S1, Supporting Information) and matches the expected symmetric behavior of charge redistribution in blocking conditions. As blocking conditions are used in this work, this can be seen as further proof of the method's accuracy.

(1) $$\begin{array}{c} \begin{array}{l} n_{\text{Li},\text{excess}}\left[\text{mol}\right]\;\;=\;\;\frac{V_{\text{Li},\text{excess}}\left[{\text{cm}}^{3}\right]\rho_{\text{Li}}\left[g\text{​}cm^{-3}\right]}{m_{\text{Li}}\left[g\text{​}{\text{mol}}^{-1}\right]}\text{​}\\ V_{\text{Li},\text{excess}}\left[{\text{cm}}^{3}\right]\;\;=\;\;c_{\text{SCL}}\left[\%\right]A\left[{\text{cm}}^{2}\right]d_{\text{SCL}}\left[\text{cm}\right] \end{array} \end{array}$$

Here *m*_Li_ is the Li molar mass, ρ_Li_ the Li density and A the electrode area. The variables *c*_SCL_ and *d*_SCL_ are the concentration change and thickness of the charged layer, respectively.

A comparison to the recorded current and its integration over time, that is the charge accumulation, is hindered by the ambiguous contributions of electronic leakage, polarization, and the very low current (nA) measured by the potentiostat as possible errors are integrated over time. Even a three‐electrode setup, which would allow accounting for the polarization and electronic leakage, would not give the true current flow from the n‐SCL to the p‐SCL as some space charge would form around the reference electrode. A dynamic analysis of the space charge formation is beyond the scope of this study.

## 3 Conclusion

In situ ellipsometry allows detection of the formation of SCL under different bias potentials. With monochromatic transient measurements, the formation of an additional layer at the interface is proven once a bias potential is applied. The correlation of ellipsometric angles and the redistributed charge is not unambiguous. A semi‐empirical model, based on a three‐layer model with a dispersion relation for each constituent of the structure, is used to fit the spectroscopic ellipsometry data and quantify the SCL formation. The suspected asymmetry is visible in the data with a wider, but less concentrated p‐SCL ((322 ± 16) nm at 1 V) compared to the thinner n‐SCL ((191 ± 19) nm at −1 V). Charges are shown to be only redistributed, as the impact of total charge redistribution is symmetric for n‐SCL and p‐SCL. These findings are a proof‐of‐concept for applying spectroscopic ellipsometry in the detection of thin, electrochemical layers with a resolution in the nanometer‐scale. The quantification of concentration changes of Li^+^ inside the SCLs can be used to parameterize and validate a wide range of theoretical models. A profound understanding of the physical extent to which the SSE can be depleted of mobile charges is fundamental to understanding possible mitigation strategies, and we believe that future research in this area can use the findings of this work.

## 4 Experimental Section

### SSE

LICGC (Ohara Inc, Japan) was used for electrochemical and optical experiments conducted in this study. The SSE had a thickness of 150 µm and was stable in the ambient atmosphere.

### Gold Electrodes

The gold current collectors were deposited using an e‐beam evaporator, Leybold 540 (Leybold, Germany), using a circular mask (radius = 7.5 mm) under high vacuum conditions (value is 3 × ​10^–7^ mbar). The deposition rate (1 Å s^–1^) and final thickness (25 nm) were controlled with an IC6000 deposition controller (Inficon, Switzerland).

### Spectroscopic Ellipsometry

An EP4 imaging ellipsometer (Accurion, Germany) was used to perform spectroscopic ellipsometry at different potentials and in situ ellipsometry was done at an AOI of 65° using a 658 nm solid‐state laser. For spectroscopic measurements, the wavelength from 360 to 1000 nm in 50 equidistant photon energy steps was adjusted using a built‐in grading monochromator, and a laser stabilized xenon arc lamp. A resting period of 2.5 h after applying the bias potential and before the spectroscopic scans was used to allow the system to reach electrochemical equilibrium.

### Sample Polarization

The samples were contacted with a gold pin‐contact on the front and a copper plate on the back. A proper contact was ensured by electrochemical impedance spectroscopy. A SP‐150 Potentiostat (BioLogic, France) was used for applying the potentials. The working electrode connected to the gold pin‐contact was set to the given voltage versus the counter electrode connected to the copper plate. The leakage currents after the aforementioned rest period of 2.5 h were consistent with the expected electronic leakage.

### XRR

A D8 Advanced X‐ray reflectometer (Bruker AXS, Germany) with a copper *K*_α_ source (wavelength 0.154 nm) and a scintillation detector was used.

### EDX

An INCAPentaFET‐x3 EDX (Oxford Instruments, UK) together with the INCA software was used to measure the EDX spectra and determine the chemical compositions.

## Conflict of Interest

The authors declare no conflict of interest.

## Author Contributions

The manuscript was written through the contributions of all authors. All authors approved the final version of the manuscript. L.K., L.C., and S.J.S. contributed equally to this work.

## Supporting information

Supporting Information

## Data Availability

The data that support the findings of this study are available from the corresponding author upon reasonable request.
