# Supplementary material for: Characterization and Quantification of Depletion and Accumulation Layers in Solid‐State Li+‐Conducting Electrolytes Using In Situ Spectroscopic Ellipsometry
Source: Adv Mater. 2021 May 6;33(24):2100585. doi: 10.1002/adma.202100585 (PMC11468716; doi:10.1002/adma.202100585)
Supplement: Supplementary file 1 — Supporting Information [file ADMA-33-2100585-s001.pdf]

© 2021 Wiley-VCH GmbH

ADVANCED MATERIALS

Supporting Information

for *Adv. Mater.,* DOI: 10.1002/adma.202100585

Characterization and Quantification of Depletion and Accumulation Layers in Solid-State Li^{+}-Conducting Electrolytes Using In Situ Spectroscopic Ellipsometry

*Leon Katzenmeier, Leif Carstensen, Simon J. Schaper, Peter Müller-Buschbaum, and Aliaksandr S. Bandarenka**

© 2021 Wiley-VCH GmbH

Supporting Information

Characterization and Quantification of Depletion and Accumulation Layers in Solid-State Li$^+$-Conducting Electrolytes Using *In situ* Spectroscopic Ellipsometry

*Leon Katzenmeier, Leif Carstensen, Simon J. Schaper, Peter Müller-Buschbaum, Aliaksandr S. Bandarenka**

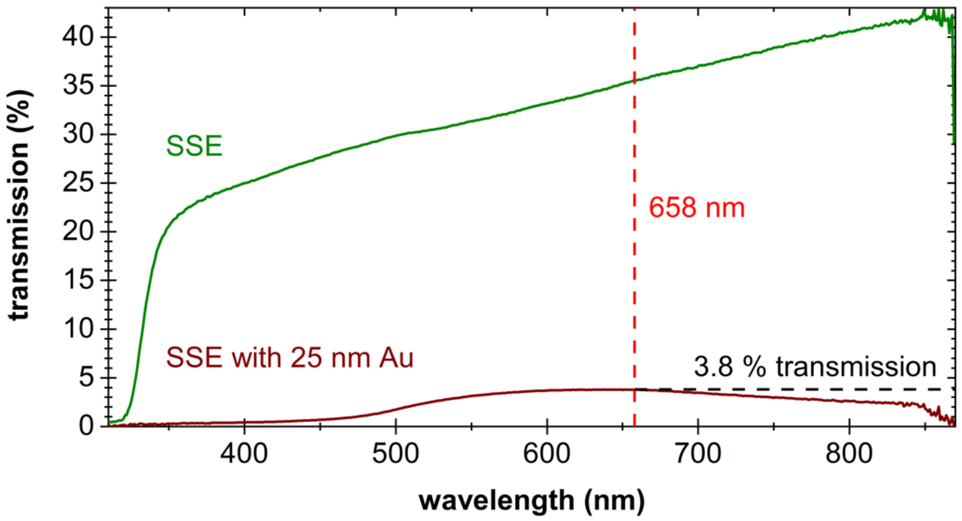

Figure S1. UV-VIS transmission spectra of the solid-state electrolyte (SSE) in green and of the SSE with 25 nm Au evaporated on both sides in dark red. The red dashed line at 658 nm corresponds to the used laser wavelength for the *in situ* ellipsometric measurements. Maximum transmission of 3.8 % (black dashed line) of the Au/SSE/Au sample is reached.

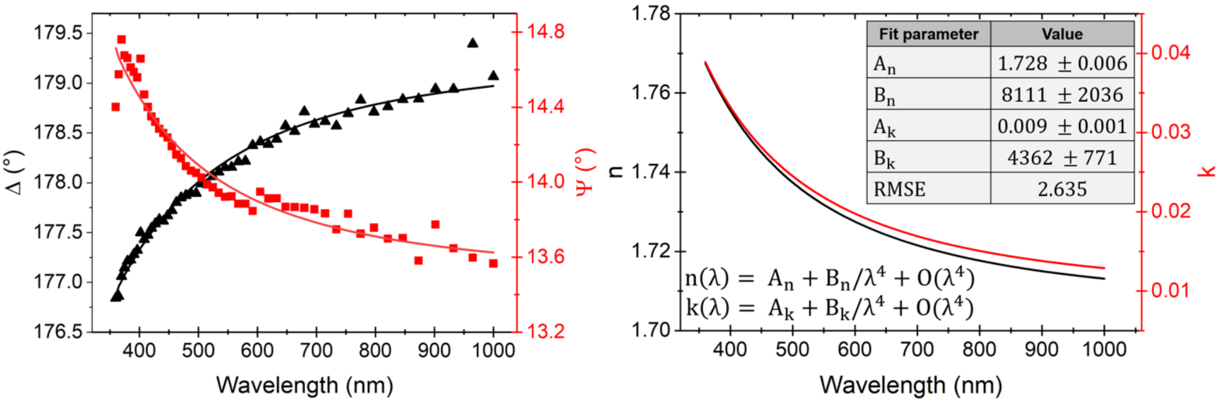

Figure S2. Spectroscopic ellipsometry measurement of the bare solid-state electrolyte. The complex diffractive index is fitted with a Cauchy distribution and used to analyze the Au/SSE/Au sample.

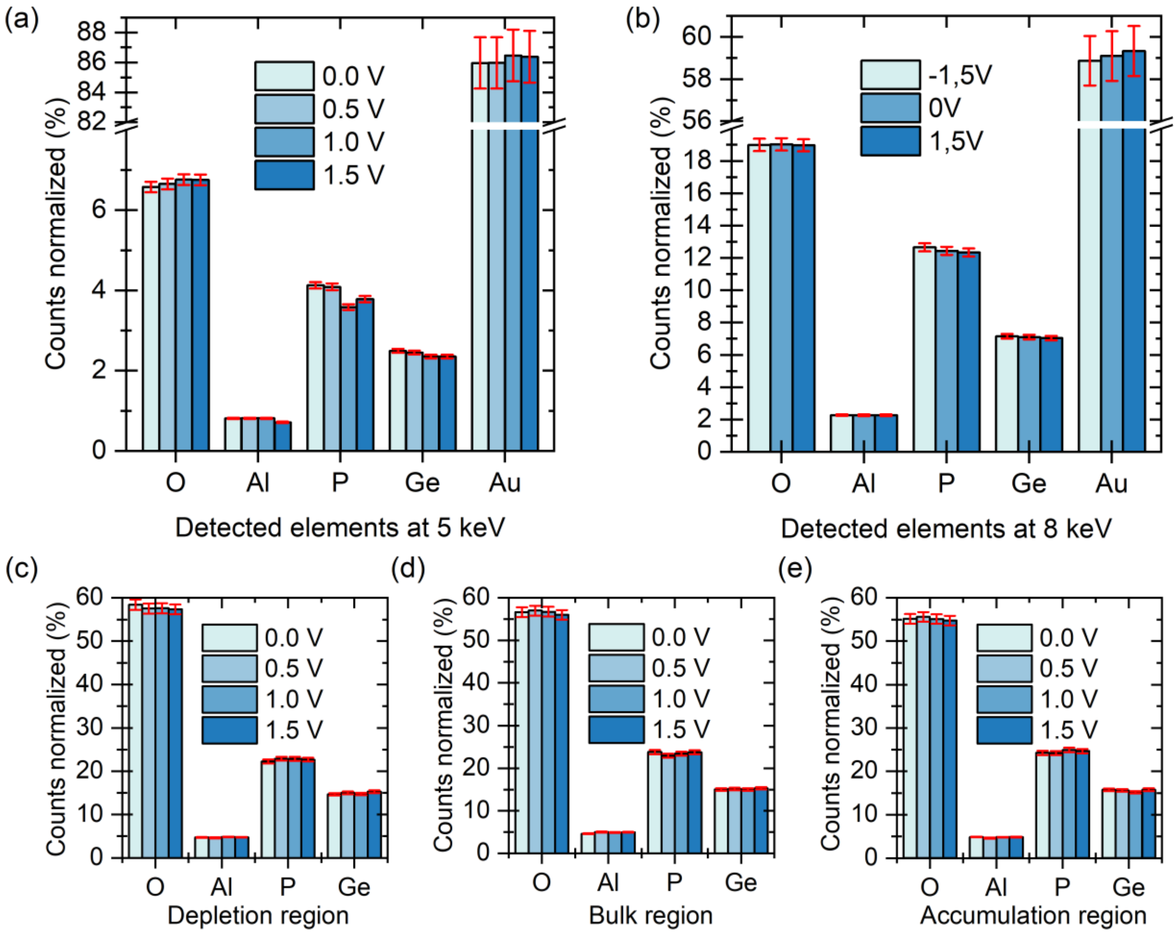

Figure S3. Energy-dispersive X-ray spectroscopy (EDX) measurements in vertical view (Au electrode detectable) at (a) 5 and (b) 8 keV for different applied potentials. Position sensitive, cross-sectional EDX measurements for applied potentials from 0 to 1.5 V of the depletion (c), bulk (d), and accumulation region (e). As there are no changes in the amount of the detected elements within the errors visible upon applied potentials up to 1.5 V, the bulk solid-state electrolyte and its anodic part are considered stable. Lithium could not be detected in these measurements.

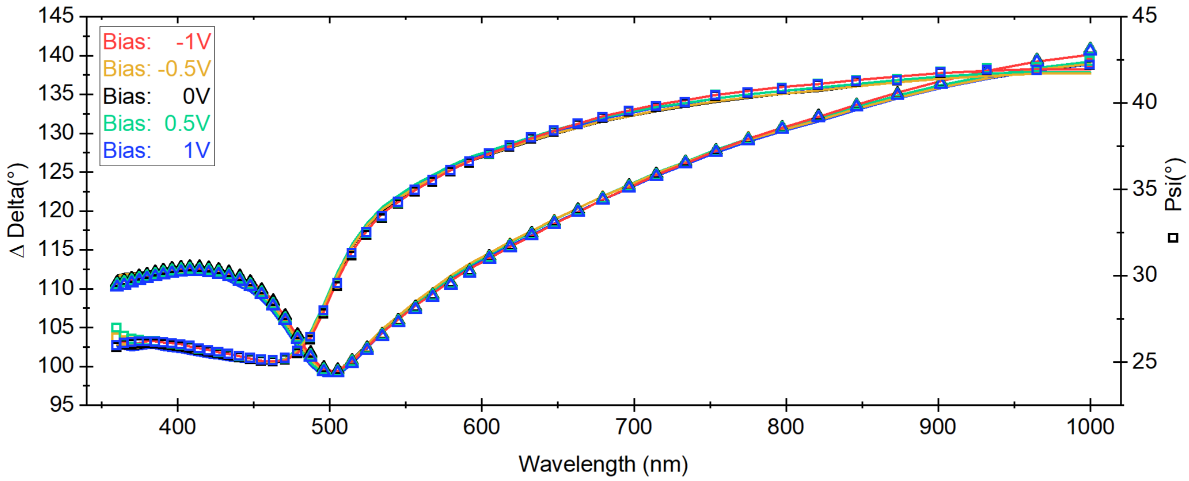

Figure S4. *In situ* spectroscopic ellipsometry measurements at applied bias potential from -1 to 1 V.

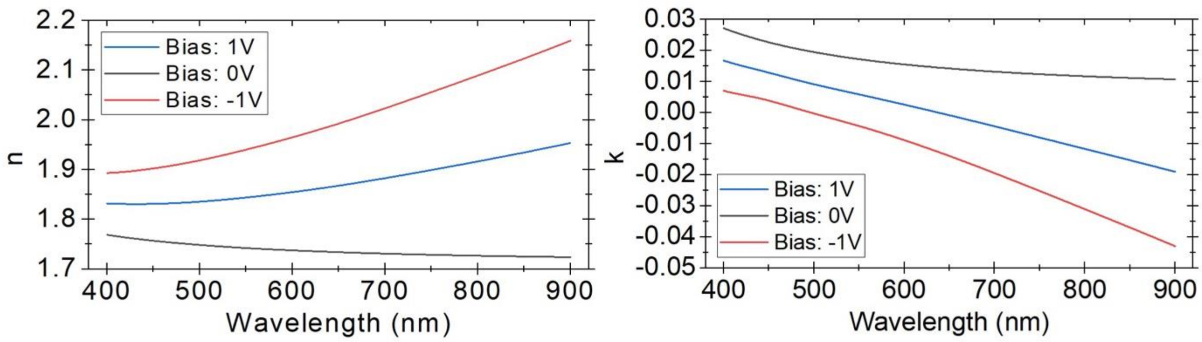

Figure S5. The optical properties of the SCL, given by the refractive index, n, and extinction coefficient, k, for three different polarization states calculated based on the Bruggemann EMA model. The 1V and -1V spectra were calculated based on a Li concentration change of -8% and +3% compared to the solid electrolyte.

Table S1. Results of the SCL fit model based on an effective medium approximation for different applied potentials.

Applied Bias [V]	-1.0	-0.75	-0.5	-0.25	0.0	0.25	0.5	0.75	1.0
SCL thickness d_{SCL} [nm]	190.7 ± 19.0	189.5 ±16.8	189.9 ±13.2	58.2 ± 9.4	21.7 ± 19.6	138.7 ± 15.6	318.7 ± 17.1	326.0 ± 13.2	321.8 ± 15.7
SCL concentration c_{SCL} [%]	-8 ± 1	-7 ± 1	-8 ± 1	-3 ± 1	-5 ± 3	2 ± 1	3 ± 2	4 ± 2	3 ± 2
N_{excess} [µmol]	2.08 ± 0.33	1.81 ± 0.30	2.07 ± 0.30	0.24 ± 0.09	0.15 ± 0.16	0.38 ± 0.19	1.30 ± 0.87	1.78 ± 0.89	1.31 ± 0.88
Fit quality (RMSE)	4.5	5.9	9.0	9.9	9.4	8.9	6.4	9.9	11.5
